# Identification and Verification of Error-Related Potentials Based on Cerebellar Targets

**DOI:** 10.3390/brainsci14030214

**Published:** 2024-02-26

**Authors:** Chang Niu, Zhuang Yan, Kuiying Yin, Shenghua Zhou

**Affiliations:** 1 Department of Electronic Engineering, Xidian University, Xi’an 710126, China; changniu1250@163.com (C.N.);; 2 Nanjing Research Institute of Electronic Technology, Nanjing 210019, China

**Keywords:** electroencephalography (EEG), error-related potentials (ErrPs), discriminative canonical pattern matching, cerebellar regions, classification method, screening method

## Abstract

The error-related potential (ErrP) is a weak explicit representation of the human brain for individual wrong behaviors. Previously, ErrP-related research usually focused on the design of automatic correction and the error correction mechanisms of high-risk pipeline-type judgment systems. Mounting evidence suggests that the cerebellum plays an important role in various cognitive processes. Thus, this study introduced cerebellar information to enhance the online classification effect of error-related potentials. We introduced cerebellar regional characteristics and improved discriminative canonical pattern matching (DCPM) in terms of data training and model building. In addition, this study focused on the application value and significance of cerebellar error-related potential characterization in the selection of excellent ErrP-BCI subjects (brain–computer interface). Here, we studied a specific ErrP, the so-called feedback ErrP. Thirty participants participated in this study. The comparative experiments showed that the improved DCPM classification algorithm proposed in this paper improved the balance accuracy by approximately 5–10% compared with the original algorithm. In addition, a correlation analysis was conducted between the error-related potential indicators of each brain region and the classification effect of feedback ErrP-BCI data, and the Fisher coefficient of the cerebellar region was determined as the quantitative screening index of the subjects. The screened subjects were superior to other subjects in the performance of the classification algorithm, and the performance of the classification algorithm was improved by up to 10%.

## 1. Introduction

In recent years, several studies showed that the cerebellum plays an important role in cognition and learning. Recent studies suggested that the cerebellum affects cognitive function by forming a wide range of neural functional circuits in specific brain regions [1]. Many research methods have been used to provide an anatomical basis for cerebellar participation in cognition, among which cerebellar circuitry is the focus of the current research. KOZIOL showed that the cerebellar connection is composed of feedforward and feedback connections between the brain and cerebellum [2]. Zaf, et al. used diffusion tensor tractography to demonstrate the existence of the cerebral–cerebellar circuit, indicating that the cortical–pontine–cerebellar pathway plays a role in regulating voluntary movement and exercise plan execution, as well as advanced cognitive, visual, and auditory functions [3]. Christophe Habas believes that “resting-state” static and dynamic functional connectivity networks are fundamental to the neocerebellum’s cognitive functions in motor and non-motor tasks [4]. Espinoza, A.I., et al. demonstrated a potential cerebellar biosignature for neurostimulation therapy to improve gait dysfunctions [5]. The above studies powerfully demonstrated the relationship between the cerebellum and cognitive activities.

In addition, many studies on cognitive functions demonstrated the cerebellum’s contributions to specific neural activities through high-density scalp EEGs without additional electrodes at the level of the neck. For example, Cebolla, A.M., et al. proposed a study on scalp EEG in a weightless state, which indicated that the cerebellum and the vestibular network’s involvement in weightlessness might support the correction signals processing necessary for postural stabilization. Furthermore, they also demonstrated the cerebellar contribution to the P300 on Earth [6,7]. Andersen, L.M., et al. proposed practical guidelines to optimize the detection of cerebellar activity with MEG and EEG [8]. Zarka, D., et al. conducted research on the EEG signals of children with attention-deficit/hyperactivity disorder (ADHD) under the go/no-go paradigm, and source analyses elucidated the relationship between cerebellar activity and the P3 component in no-go trials [9]. Reyes, S.A., et al. used source localization techniques to determine that both sides of the cerebellum are associated with the 40 Hz auditory steady-state response to specific frequencies [10]. Stanacak, A. and Fallon, N suggested that when laser stimuli are paired with negative emotional images, activations in the cerebellum are detected [11]. Elshoff, L., et al. used dynamic imaging of coherent sources (DICSs) to analyze scalp EEG data during epileptic seizures and demonstrated that the cerebellum was one of the sources of epileptic seizures [12].

In short, mainstream EEG and BCI research has seldom utilized cerebellar scalp regional electrodes and their signal features. If the central nervous system signals represented in the cerebellar region could be deeply analyzed, it might be possible to significantly enhance the performance of the BCI.

Event-related potentials (ERPs) are important EEG-evoked potentials [13], and they have been widely used as a method for decoding brain signals due to their low cost and fast response. In particular, when a participant performs a task with a false response, an error-related potential can be recorded on the scalp, which is referred to as an ErrP [14]. Studies showed that the ErrP is generated under specific task conditions. For example, a responsive ErrP occurs when subjects are asked to respond as quickly as possible (e.g., a key press response task) [15,16,17], and a feedback ErrP occurs when a subject is aware of errors based on task feedback [18]. An interactive ErrP occurs when the subject interacts with the machine and the machine executes the wrong instruction [19], and an observational ErrP occurs when the subject observes an error in a machine or external device [20,21]. At present, cerebellar electrodes are rarely applied to online BCI systems. Therefore, this study attempted to introduce cerebellar electrode signals and features into BCI systems to improve their classification performance.

The system performance of an ERP-BCI depends largely on classification algorithms. Researchers have conducted research and comparisons to discover a better ERP classification algorithm. Krusienski, et al. compared five classification algorithms for P300 features, including Pearson’s correlation method (PCM), linear discriminant analysis (LDA), stepwise linear discriminant analysis (SWLDA) [22], linear support vector machine (LSVM), and Gaussian kernel support vector machine (GSVM), and they demonstrated that SWLDA and LDA have obvious advantages over other algorithms [23]. Unlike Krusienski, Aloise applied the above five algorithms to non-eye-movement-dependent P300-BCI and found that there was no significant difference in the performances of these algorithms [24]. When proposing the shrinkage linear discriminant analysis (SKLDA) [25] algorithm for single-trial ERP classification, Blankertz, et al. compared the remaining LDA and SKLDA algorithms to demonstrate the superiority of SKLDA. Zhang, et al. proposed that the spatial–temporal discriminant analysis (STDA) algorithm is superior to LDA, SWLDA, and SKLDA. Lawhern, et al. used the EEGNet method to classify ERP features, such as P300 and error-related negativity (ERN), confirming that the algorithm is suitable for intra-individual and cross-individual classification [26]. In 2018, Xiaolin, et al. from Tianjin University proposed a discriminative typical pattern-matching algorithm (DCPM) for single-trial ErrP classification [27]. In short, in the field of EEG data classification, quantitative evaluation, and screening of subjects, EEG signals and characteristics of the cerebellar region are rarely applied. The performance of online ErrP-BCI systems is limited by individual differences in EEG data and a low signal-to-noise ratio [28], and it is currently rare in engineering applications.

In event-related-potential-based brain–computer interfaces (ErrP-BCI), calibrated data can be used to classify an individual’s performance online, and the outcomes of the classification algorithm can be directly assessed. Specifically, In ErrP-BCI, misbehavior can arise from a variety of mental processing situations. As a result, some error trials cannot be recognized. In online ErrP-BCI, the algorithm can only detect and classify “complete errors” [29] in trials where the participant immediately realized they had made an error. Other error trials cannot be recognized by the EEG data classification algorithm and also cause reverse interference with the classification results. Therefore, it is necessary to use other methods for the quantitative evaluation of subjects to screen out subjects with better performance in online ErrP-BCI.

This study made improvements to the original discriminant typical pattern matching algorithm (DCPM) and optimized the process of data training and model construction of the algorithm. The improved DCPM was compared with the original DCPM to demonstrate its superiority. Furthermore, a correlation analysis was conducted in different brain regions features to quantitatively assess the actual performance of subjects in ErrP-BCI, which helped us to find excellent subjects to enhance the practical performance of BCI.

## 2. Materials and Methods

### 2.1. Subjects and Experimental Design

Thirty non-disabled volunteers, aged between 22 and 40 years, participated in this study. Twelve of them were female and eighteen were male. None of them had color blindness or sensory impairment. All of them were students and teachers of image interpretation majors in a certain university.

All participants signed a written informed consent form, and the experiment was reviewed and approved by the Ethics Committee of the Brain Hospital affiliated with Nanjing Medical University.

The feedback induction formula was used to induce the ErrP of the subject by displaying the judgment result in real time after the subject made a judgment. Thus, all four types of errors mentioned in the introduction were converted into “complete errors” to facilitate the correlation analysis of the quantitative evaluation features of the subjects based on the balance accuracy of the classification algorithm.

The picture judgment task was adjusted for picture difficulty and reaction time to induce the subjects to produce sufficient error trials. The experiment was divided into three sections, with six groups in each section and 50 trials in each group. There were 900 trials for each participant, and each trial included a self-assessment by the participant regarding their judgment. The behavioral information confirmation plan was as follows:

Only the trials in which the participant believed they made an error judgment and they did were considered error trials. Conversely, when the participant believed they made a correct judgment and they did, that trial was considered a correct trial.

This behavioral information was used to mark the correct and incorrect trials in the training set.

Using the calibrated optical image slices as the stimulus library, according to the students’ daily teaching and interpretation content collection, the slice size was unified to 500 × 500 pixels and divided into two categories: pictures with aircraft and pictures without aircraft. In the image library, 200 slices were used for each category. All slices were blurred and noise was added to increase the recognition difficulty.

**Figure 1:**
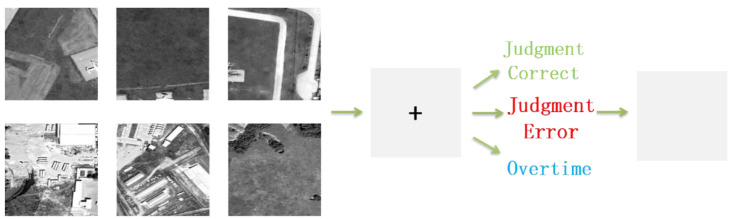
The process of each trial of experiments.

The experimental process for each trial is illustrated in Figure 1. The experimental procedure is listed from left to right in Figure 1. Initially, the image slice to be identified was presented for 1 s (the six images on the far left of Figure 1 are examples), after which the screen switched to a black cross that lasted for 0.5 s. Participants were required to make a judgment on whether the image contained an airplane target and press the corresponding key within 1.5 seconds before the black cross disappeared. Afterward, the screen would provide feedback on whether the judgment was correct. If the participant did not make a judgment within 1.5 seconds, the screen would indicate a timeout mark. Lastly, a white screen appeared for 0.5 s to prevent the ErrP component from overlapping with the visual ERP induced by the next image (the far right of Figure 1).

### 2.2. Data Acquisition and Pre-Processing

**Figure 2:**
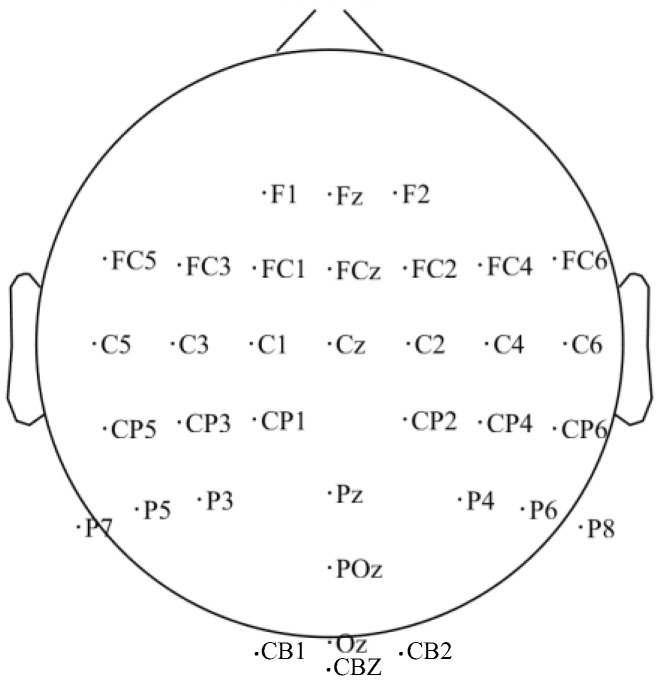
The electrode position diagram selected for the experiment.

A 71-channel EEG acquisition cap (Neuracle Technology Co., Ltd., Changzhou, China) was used, which included 69 fixed electrodes, two active electrodes, one reference electrode in the CPz position, and one ground electrode in the AFz position. The electrodes were placed according to the 10–20 international standard lead system (Figure 2). In this experiment, 35 electrodes, including cerebellar electrodes CB1, CBZ, and CB2, were selected for data acquisition (Figure 2), and the sampling frequency of the EEG data was 1000 Hz.

To eliminate the interference from electromyography (EMG) and electrooculography (EOG) to some extent, this study utilized three methods. First, we set up a camera synchronized with the EEG data acquisition at the experimental site (as noted in the informed consent form) to record the subjects’ overt movements during the experiment, and the corresponding EEG data were removed; second, during preprocessing, portions that were artifacts were manually excluded; third, subjects were encouraged to minimize blinking during the task.

In the pre-processing process, the signal was first filtered by a 1–30 Hz bandpass Butterworth filter, and the average reference of the whole brain was used. The moment when the participant pressed the key was at 0 milliseconds (the same as below). The EEG data from −500 ms to 500 ms was intercepted for each trial, and the data from −500 ms to 0 ms was taken as the baseline for correction. EEG data of 0~500 ms were processed with a zero-mean disposal.

### 2.3. Improved DCPM

An ErrP is a weak ERP with an amplitude of approximately 3~10 μV [30], and the system performance of an ERP-BCI depends largely on the classification algorithm. In recent years, the mainstream algorithms used in the academic community for the classification of ErrP have been SVM; LDA; and their improved algorithms EEGNet, DCPM, etc. [31]. Previous studies showed that the DCPM algorithm performs significantly better than the other algorithms in ErrP classification.

The original DCPM algorithm was divided into the following steps: first, discriminative spatial patterns (DSPs) were used to suppress the common-mode noise in the EEG, and canonical correlation analysis (CCA) was used to enhance the EEG features. Finally, different decoding templates were constructed according to the coding strategies of different stimulus paradigms to improve the signal-to-noise ratio of EEG signals and the classification and recognition efficiency of signal features.

The DCPM algorithm divided the data into training and test sets. During the training process of the classification model, some model training parameters were not adjustable, which could limit the performance of the classification algorithm.

**Figure 3:**
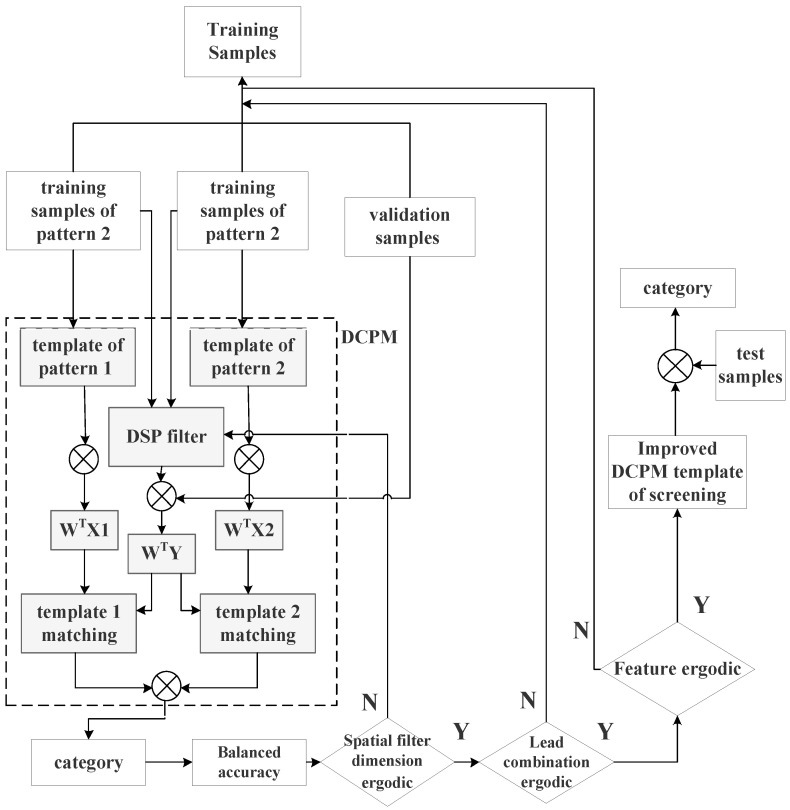
The flowchart for improved DCPM classification algorithm.

This paper proposes the following improvement scheme for the DCPM algorithm (Figure 3).

The process of the improved DCPM algorithm in Figure 3 is described as follows:

(1) Five-fold cross-validation was used to divide the training set into five subsets; one of the five subsets was used as the verification subset, and the others were used as the training subset. The training subset was used to train the model based on the currently selected parameter combination, and the verification subset was used to verify and calculate the balanced accuracy of the model. This process was repeated five times, and the balanced accuracy of the model obtained from each repetition was averaged as the performance index under the current parameter combination. The optional parameters included the lead, spatial filter dimension, and extracted pattern-matching features.
(2) The lead screening was conducted based on the Fisher separability measure, and suitable electrodes were screened by traversal.
(3) A spatial filter was constructed based on a discriminant spatial pattern and performed traversal selection on the dimensions of the spatial filter.
(4) After the input of the verification set, pattern matching was performed, and the linear correlation, spatial distance, and canonical correlation analysis were calculated.
(5) The calculated features were permuted and combined, and the model prediction was performed. The evaluation function was defined as the balance accuracy. The output value of the evaluation function was recorded. The balanced accuracy was the mean value of the proportion of correctly predicted samples in each category. The formula was as follows:

(1) $$balanced\_accuracy=\frac{1}{k}\sum_{i=1}^{k}\frac{TP(i)}{S_{i}}$$

where *k* is the class, *TP* is the number of true positive trials, and *S_i_* is the number of samples in class *i*. When the dataset was balanced, the two performance measures of the balanced accuracy and accuracy were equivalent. When the dataset was unbalanced, the balanced accuracy considered both positive and negative classes, which could better describe the performance of the classifier than the accuracy.

(6) After the above cycles were completed, the lead selection scheme, spatial filter dimension, and feature selection scheme that maximized the evaluation function were searched for and saved to the template.
(7) A new template was used to recognize the test set and record the classification results.

### 2.4. Screening Method for Excellent ErrP-BCI Subjects

This paper proposes a screening method for excellent ErrP-BCI subjects based on cerebellar target characteristics and is used to screen individuals with excellent performance of the DCPM algorithm in an online ErrP-BCI system.

In this study, the experimental paradigm given in Section 2.1 was used to obtain slices of two kinds of EEG data, and the Pearson correlation coefficient, F-score, Fisher separability measure, and other indicators were calculated between the two kinds of data under different electrodes. A correlation analysis was conducted with the evaluation function results of the online classification algorithm in the ErrP-BCI system. Then, we obtained the optimal quantitative evaluation index of the ErrP online recognition ability and quantitatively described the possible influence of individual differences on the ErrP-BCI system. By comparing the evaluation indices, the subjects could be screened, and the actual performance of the ErrP-BCI system could be optimized.

(1) Evaluation index based on the Pearson correlation coefficient: This evaluation index is used for the quantitative assessment of the representation strength of ErrP at a particular lead.

(2) $$1-\left|corr(x_{i},x_{j})\right|$$

where *corr* is the Pearson correlation coefficient, and *x_i_* and *x_j_* are the EEG data collected from a single lead for correct and error trials, respectively.

(2) Evaluation index based on the F-score [32]: The F-score is a feature importance evaluation criterion based on inter-class and intra-class distance, which can effectively measure the feature in the realization of a binary classification problem. After calculating the F-score of each sampling point of the EEG data, the mean and maximum values of each lead were utilized to calculate the evaluation indexes:

(3) $$\begin{array}{l} F_{i}=\frac{{\left({\bar{x}}_{i}^{+}-{\bar{x}}_{i}\right)}^{2}+{\left({\bar{x}}_{i}^{-}-{\bar{x}}_{i}\right)}^{2}}{\frac{B_{n+}}{n_{+}-1}+\frac{B_{n-}}{n_{-}-1}}\\ B_{n+}=\sum_{k=1}^{n_{+}}{\left(x_{k,i}^{+}-{\bar{x}}_{i}^{+}\right)}^{2}\\ B_{n-}=\sum_{k=1}^{n_{-}}{\left(x_{k,i}^{-}-{\bar{x}}_{i}^{-}\right)}^{2} \end{array}$$

where *n^+^* and *n^−^* are the numbers of two types of samples; $\bar{x}$, ${\bar{x}}_{i}^{+}$, and ${\bar{x}}_{i}^{-}$ represent the mean of the i-th feature on the whole dataset, the mean of the positive dataset, and the mean of the negative dataset, respectively. $x_{k,i}^{\;+}$ and $x_{k,i}^{\;-}$ represent the eigenvalues of the i-th feature of the k-th sample point in the positive and negative classes.

(3) Evaluation index based on the Fisher reparability measure: After calculating the Fisher index value of each sampling point, the mean and maximum values of each lead were utilized to calculate the evaluation indexes:

(4) $$\frac{\left|u1-u2\right|}{s1+s2}$$

where *u*1 and *u*2 are the mean values of the error and correct trials at the sampling point, respectively, and *s*1 and *s*2 are the variances of the error and correct trials at the sampling point, respectively.

## 3. Results

**Figure 4:**
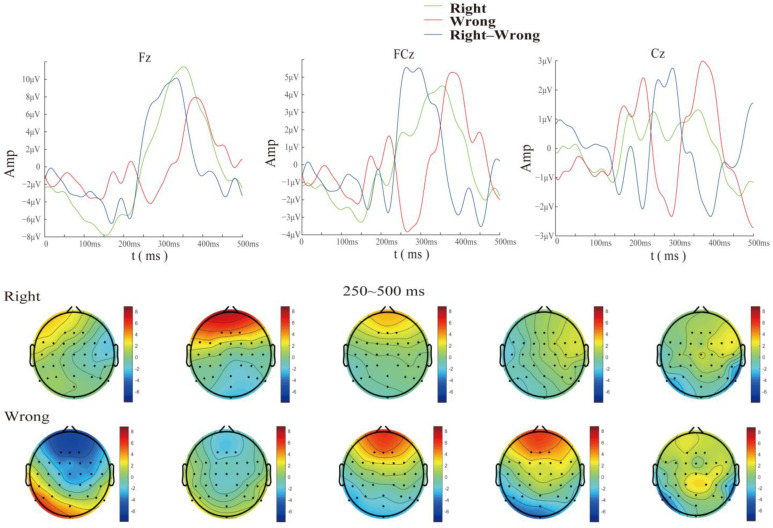
Time domain superimposed average map and brain topographic map (the first column shows the time-domain superimposed average waveform for three different electrodes, while the second and third columns show the brain topographic maps corresponding to correct and error trials, respectively, starting from 250 ms and plotted every 50 ms).

First, according to the comprehensive analysis of the individual’s brain topographic map and time-domain waveform diagram (Figure 4), it was found that the error trials between different subjects had obvious commonalities. After the superposition and averaging of the error trials, it can be clearly seen that the time-domain waveform diagrams generally exhibit a more obvious negative wave around 200–400 ms after the button was pushed, followed by a positive reversal, which is a typical ERN time-domain representation. In the brain topographic map, it can be seen that the significant difference area was located in the frontal lobe and the central area, and the difference time was approximately 250 ms after the button was pushed.

**Figure 5:**
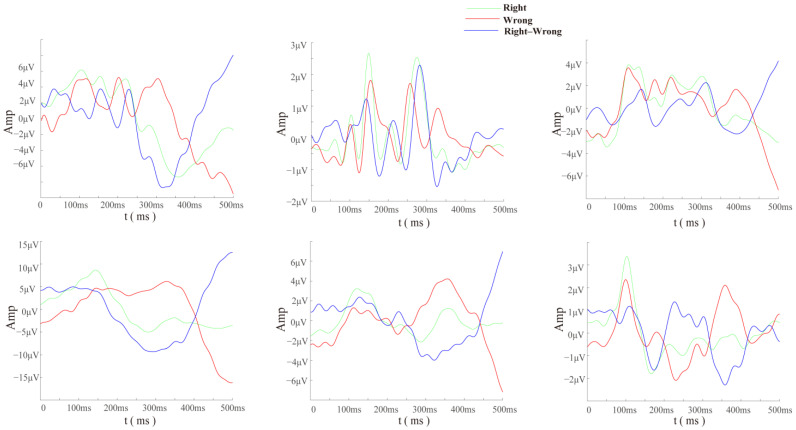
Subjects of cerebellar CBz lead EEG data superimposed average figure.

In addition, we selected six subjects with the most incorrect trials out of a total of 30 subjects for waveform analysis since their data were relatively balanced. Through the time-domain waveforms of error trials from different subjects’ central cerebellar electrodes (CBz) (Figure 5), it can be seen that the EEG data at the cerebellar electrode presented obvious specific components in the case of misjudgment. This indicates that to some extent, the cerebellum is involved in higher cognitive processing of the brain.

**Figure 6:**
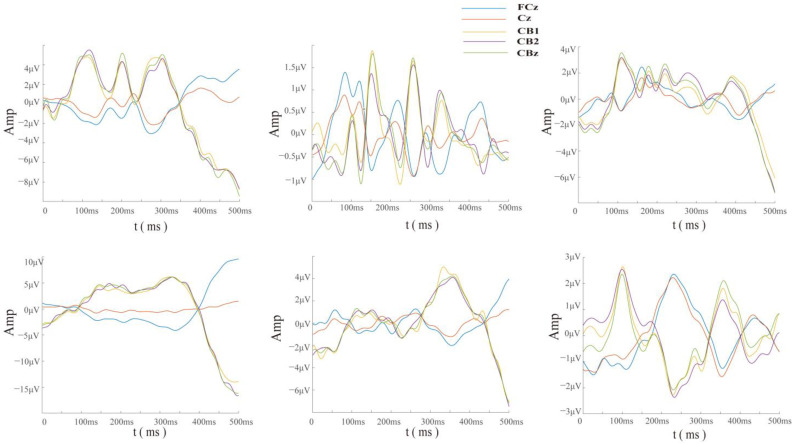
The average comparison of FCZ, CZ, CBZ, CB1, and CB2 of 6 subjects.

For each participant, electrodes FCz and Cz, which show significant differences in the specificity of cerebellar electrodes and ErrP, were compared (Figure 6). It can be seen that the direction of the difference wave of the cerebellar electrode was opposite, and the amplitude was higher. This suggests that the cerebellum could elicit more significant error-related potential components than the cerebral cortex under the same conditions and within the same subjects.

**Figure 7:**
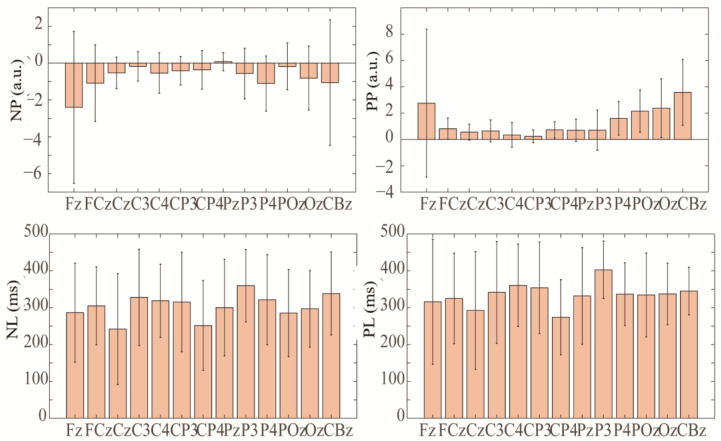
Dispersion of the ERN and Pe amplitudes and latencies across some channels.

Figure 7 shows the distributions of ERN and Pe amplitudes and latencies for some channels. As can be seen, the larger Pe (3.57 ± 2.49 a.u.) occurred on CBZ, with a latency of 345 ± 64 ms. The electrodes Fz, FCz, P4, Oz, and CBz showed larger ERN peaks of −2.4 ± 4.7, −1.8 ± 2.0, −1.1 ± 1.4, −0.8 ± 1.7, and −1 ± 3.4 a.u., respectively, with latencies of 286 ± 134, 305 ± 105, 321 ± 122, 296 ± 103, and 338 ± 112 ms. Electrode Cz showed a shorter ERN latency of 241 ± 150 ms, with a peak of −0.5 ± 0.85 a.u. Among them, electrode Fz had larger ERN and Pe peaks at the same time, but there was a larger standard deviation due to individual differences. The time-domain characteristics of ErrP in the cerebellar region were obvious, which may be helpful for the identification and verification of ErrP.

**Figure 8:**
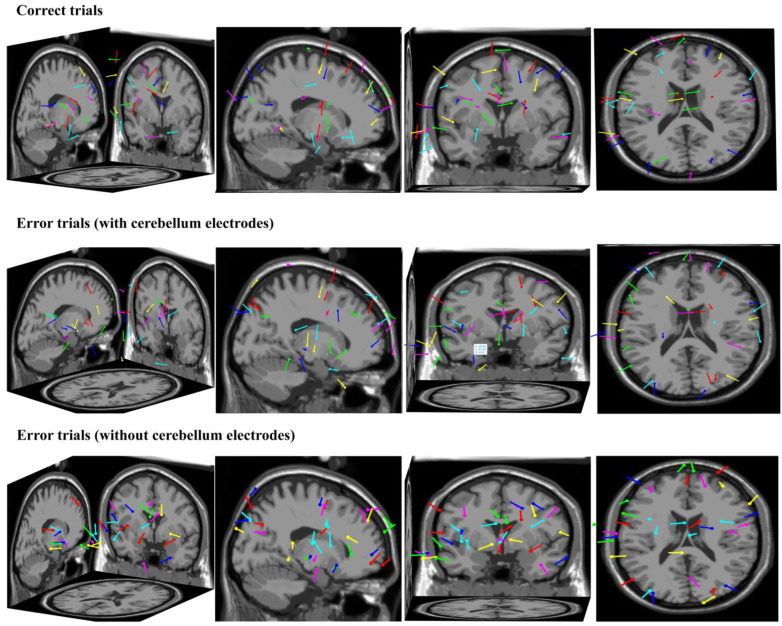
The dipole localization results for correct and error trials.

From the perspective of the source analysis, we used ICA and Dipfit to perform dipole localization analysis on the dataset collected in this study. The data-processing steps were as follows: (1) we took the button press moment of the participant as time zero and we extracted the EEG data from −200 ms to 500 ms for a single trial; (2) trials that went overtime or had obvious artifact interference were excluded; (3) the remaining trials were divided into correct and error trials, which were then subjected to independent component analysis and dipole localization, respectively, yielding the localization results shown in Figure 8. The first row shows the dipole localization results for correct trials from different perspectives, while the second row and third row show the dipole localization results for error trials with and without cerebellum electrodes, respectively. It can be observed that in the dipole localization results for error trials with cerebellum electrodes, there was a certain degree of activation in the cerebellar region (dipole 1—X: −59, Y: −39, Z: −13; dipole 2—X: −25, Y: −27, Z: −13), which was not present in the dipole localization results for correct trials and error trials without cerebellum electrodes. This further illustrates the representation of cerebellar activity at the neck scalp electrodes during error-related potential activation.

**Figure 9:**
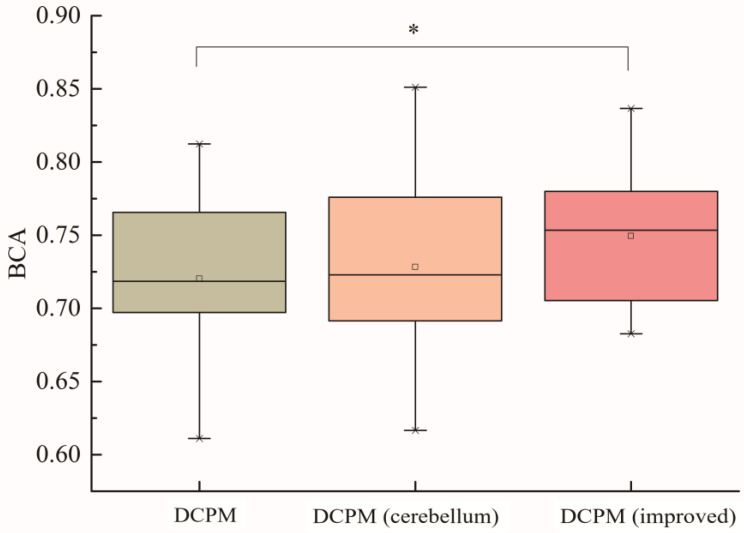
Comparison of algorithm improvement results (‘*’ represents *p* < 0.05).

Figure 9 is a boxplot, which is mainly used for observing the overall distribution of data. The boxplot can provide a visual display of a dataset by calculating and displaying statistical measures, such as the median, 25th percentile, 75th percentile, upper limit, and lower limit. An analysis of variance (ANOVA) test and t-test were used to statistically test the results. For the 30 subjects in this study, each subject selected training samples from offline data and selected 900 test samples for training, verification, and testing. The average balanced accuracy of the 30 subjects was calculated, and the classification results of the original DCPM algorithm, the DCPM algorithm after the introduction of cerebellar electrodes, and the improved DCPM algorithm (with cerebellum electrodes) are shown in Figure 9. From the test results, it can be seen that there was no significant difference between the calculation of DCPM features after adding cerebellar electrodes and the original DCPM algorithm, while there was a significant difference between the improved DCPM algorithm and the original DCPM algorithm, and the average balance accuracy of the improved DCPM (with cerebellum electrodes) algorithm was about 5% higher than that of the original DCPM algorithm.

In addition, this study used the DCPM, improved DCPM, SVM, and SKLDA to process the dataset collected in this study simultaneously and obtained the performance comparison table for the algorithms (as shown in Table 1). The table shows that compared with traditional pattern recognition algorithms, the DCPM and the improved DCPM algorithms significantly improved the true positive rate (TPR) and overall performance.

Finally, the evaluation indices of the screening methods for excellent ErrP-BCI subjects in each brain region were compared. The correlation coefficient was calculated with the balance accuracy of the ErrP-BCI data classification under the feedback condition designed in this study and the results of the 30 subjects were averaged. Table 2 presents the results. 

First, through a horizontal comparison, it was found that the Fisher coefficient, whether its mean or maximum value, was highly correlated with the actual performance of the subjects in ErrP-BCI. Second, through a longitudinal comparison, it was also found that the characteristics of the cerebellar region had the highest correlation coefficient with the actual performance of the subjects in the ErrP-BCI across all brain regions.

**Figure 10:**
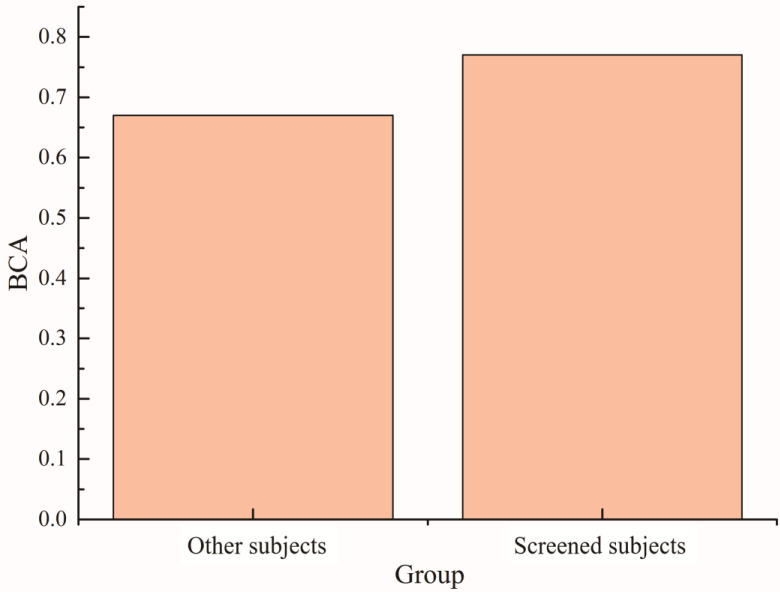
The accuracy of classification balance in ErrP-BCI system was compared between the subject screening group and the other groups.

Based on the above results, the maximum Fisher coefficient value after the superposition of the two types of training data from the cerebellar region electrode was used as the evaluation index. The subjects were screened using a threshold of 0.15. The 30 subjects were divided into two groups to compare their balance accuracy in the feedback ErrP-BCI system. The results are shown in Figure 10. The classification balance accuracy of the screening group in the ErrP-BCI system was approximately 10% higher than that of the other subjects. The effectiveness of this screening method is also illustrated.

## 4. Discussion

In this study, an experimental paradigm based on image interpretation was designed to collect EEG data on feedback-error-related potentials. At the same time, in the process of EEG acquisition, three electrodes were added below the occipital region to collect EEG signals near the cerebellum to study the relationship between the cerebellum and incorrect behavior. In this experiment, we recruited 30 subjects to participate. Each subject provided 900 trials of EEG data; 500 ms of EEG data were obtained after each trial for subsequent classification algorithms and excellent subject-screening methods.

During the analysis of the time-domain waveform graphs and brain topographic maps, we found a typical representation of the ErrP signal in the cerebellar region: almost all subjects’ ErrP peaks occurred between 250 and 400 ms after the wrong behavior, and compared with the ErrP signal representation of the electrodes in the frontal and parietal regions, the amplitude was stronger and the direction was opposite. This can be considered lateral evidence of cerebellar involvement in cognitive activity. Additionally, studies showed that cerebellar electrode signals could be used to improve P300 [33] and ERN [34] classification algorithms. Therefore, we reasonably speculate that the EEG data of the cerebellar region has a positive effect on the classification algorithm and excellent subject screening. We used a dipole source analysis method based on Dipfit to obtain two dipoles located in the cerebellar region when error feedback occurs, which supports the conclusions above.

With respect to classification algorithm improvement research, this study improved the original DCPM classification algorithm in the process of data training and model construction, and the verification set was introduced. The lead selection was based on the Fisher coefficient between the average of the correct and wrong trials of each lead and used a triple-nested loop to optimize the classification model parameters. The essence of the improved algorithm involves exchanging a more effective ErrP classification template for specific subjects at the cost of increasing the number of training calculations of the classification model. However, in an online ErrP-BCI, the classification model is often trained in advance. Therefore, the influence of an increase in the number of training calculations of the classification model is negligible.

In terms of excellent subject screening, we selected the Pearson correlation coefficient, F-score, and Fisher coefficient as the evaluation indices to be studied. Then, the correlation between the evaluation indices of each brain region and the balance accuracy of the improved DCPM algorithm was analyzed to determine the optimal quantitative indicators of the actual performance of different subjects in the ErrP-BCI system.

## 5. Conclusions

This study investigated error-related potentials. The correlation between the EEG data of the cerebellar region and the feedback-error-related potential was explored, and the specific characterization of the potential in the cerebellar region was summarized. The ErrP peak of the EEG data in the cerebellar region was approximately 250~400 ms after the error behavior, the amplitude was stronger, and the direction was opposite compared with the ErrP signal characterization of the electrodes in the frontal and parietal regions. In addition, we propose an improved DCPM EEG data classification algorithm. Through comparative experiments, it was found that the balance accuracy index of the improved classification algorithm for all 30 subjects was approximately 5~10% higher than that of the original DCPM classification algorithm, and the improved DCPM was superior to traditional pattern recognition algorithms (SVM, SKLDA, etc.). Only including the cerebellar electrode in the EEG data classification showed no significant improvement in balance accuracy, as shown in Figure 9. Then, we utilized a dipole source localization method based on Dipfit, which clarified that certain regions of the cerebellum were activated following an error in behavior. It is worth mentioning that the improved DCPM algorithm was used to optimize the parameters and increase the cerebellar electrode EEG data to obtain a more suitable classification model for the subjects. This method can be applied to improve other EEG data classification algorithms. Finally, in terms of screening excellent subjects, this study found that the Fisher coefficient in the cerebellar region had a high correlation with the balance accuracy of the improved DCPM algorithm. Therefore, this paper proposes a screening method based on the Fisher coefficient features of cerebellar targets. The classification performance of the selected subjects was compared with that of other subjects. The comparison analysis showed that the subjects selected by the cerebellum-related target features had a greater improvement in ErrP-BCI classification performance than other subjects.

It should be noted that the cerebellar target features discussed in this paper were characteristics of the EEG signals collected from the scalp corresponding to the cerebellum. Whether these features were caused by cerebellar neural activity requires further research and source analysis. However, our work at least suggests that there may be a potential cerebellar biosignature that positively influences the classification of ErrP and the selection of excellent subjects. We will expand the dataset in subsequent studies and conduct source analysis on the EEG data to further determine the impact of the cerebellum on cognitive functions.

## Figures and Tables

**Table 1:** The classification results of different algorithms (with cerebellar electrodes) on the ErrP dataset collected in this study.

	SVM	SKLDA	DCPM	Improved DCPM
Average accuracy	0.705	0.735	0.729	0.743
Average true positive rate	0.536	0.582	0.740	0.809
Average balanced accuracy	0.648	0.684	0.732	0.766

**Table 2:** The correlation between the indices of different brain regions and accuracy of balance.

	Corr	F-Score (Mean)	F-Score (Max)	Fisher (Mean)	Fisher (Max)
F	0.219	0.462	0.433	0.464	0.453
C	0.392	0.518	0.292	0.538	0.307
P	0.002	0.428	0.53	0.428	0.520
O	0.17	0.53	0.54	0.526	0.539
CB	0.205	0.689	0.72	0.694	0.731

## Data Availability

The data presented in this study are available on request from the corresponding author due to privacy reasons.
